# Supplementary figures and images for: IGSF11 is required for pericentric heterochromatin dissociation during meiotic diplotene
Source: PLoS Genet. 2021 Sep 7;17(9):e1009778. doi: 10.1371/journal.pgen.1009778 (PMC8448346; doi:10.1371/journal.pgen.1009778)

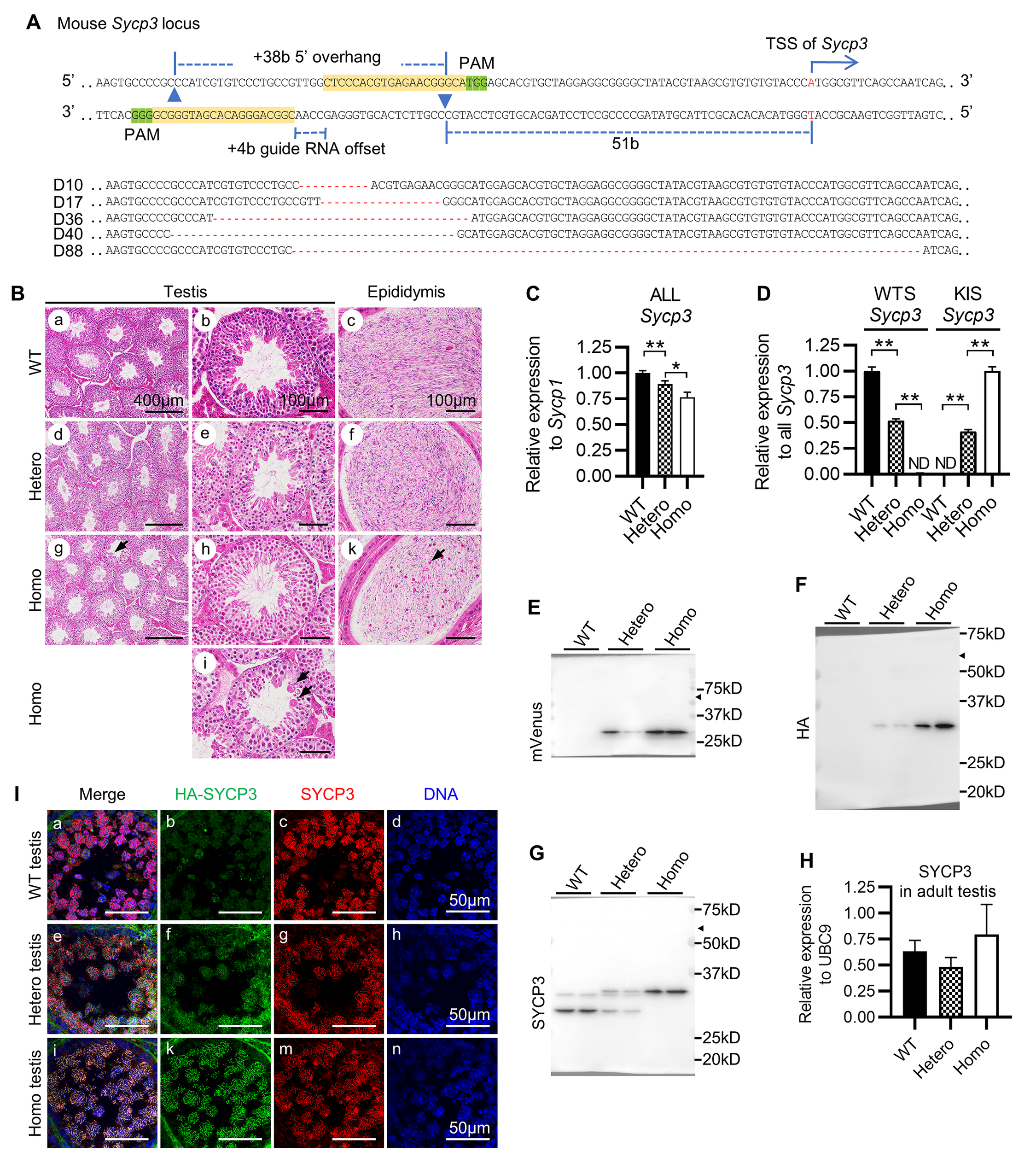

Supplement: S1 Fig — (A) Design of guide RNAs for Sycp3 targeting. Guide RNA recognition sequences and protospacer-adjacent motif (PAM) are shown on yellow or green background, Cas9 cleavage sites were indicated by triangular arrowheads. The downstream cleavage site is 51 bases upstream the transcription start site (TSS) of Sycp3. Indels that caused by Cas9-mediated double strand breaks in targeted mouse embryos as confirmed by sequencing. Abbreviations (the same as below): D10, Deletion 10 base. (B) Histological analysis of VPHS transgenic testes. Arrowheads indicate degenerated cells occasionally found in VPHS homozygote. (C) Expression of total Sycp3 transcript (ALL Sycp3) in adult testis of different VPHS genotypes. Biological repeats = 3, Technical repeats = 3. (D) Expression of wild type (WTS) and knockin (KIS) Sycp3 transcript in adult testis with different VPHS genotypes. Biological repeats = 3, Technical repeats = 3. (E-H) Protein expression of VPHS transgene in adult testis (E-G). Triangle star indicates the region if non-cleavage mVenus-P2A-HA-SYCP3 (59kD) appear. Relative SYCP3 levels were normalized to UBC9 (H). n = 2 animals/genotype. (I) Detection of transgenic HA-‍SYCP3 in 17 dpp seminiferous tubules of different VPHS genotypes. Values and error bars are mean and SD. *, P < 0.05; **, P < 0.01 by Student’s t test. ND, not detected. (TIF) [file pgen.1009778.s001.tif]

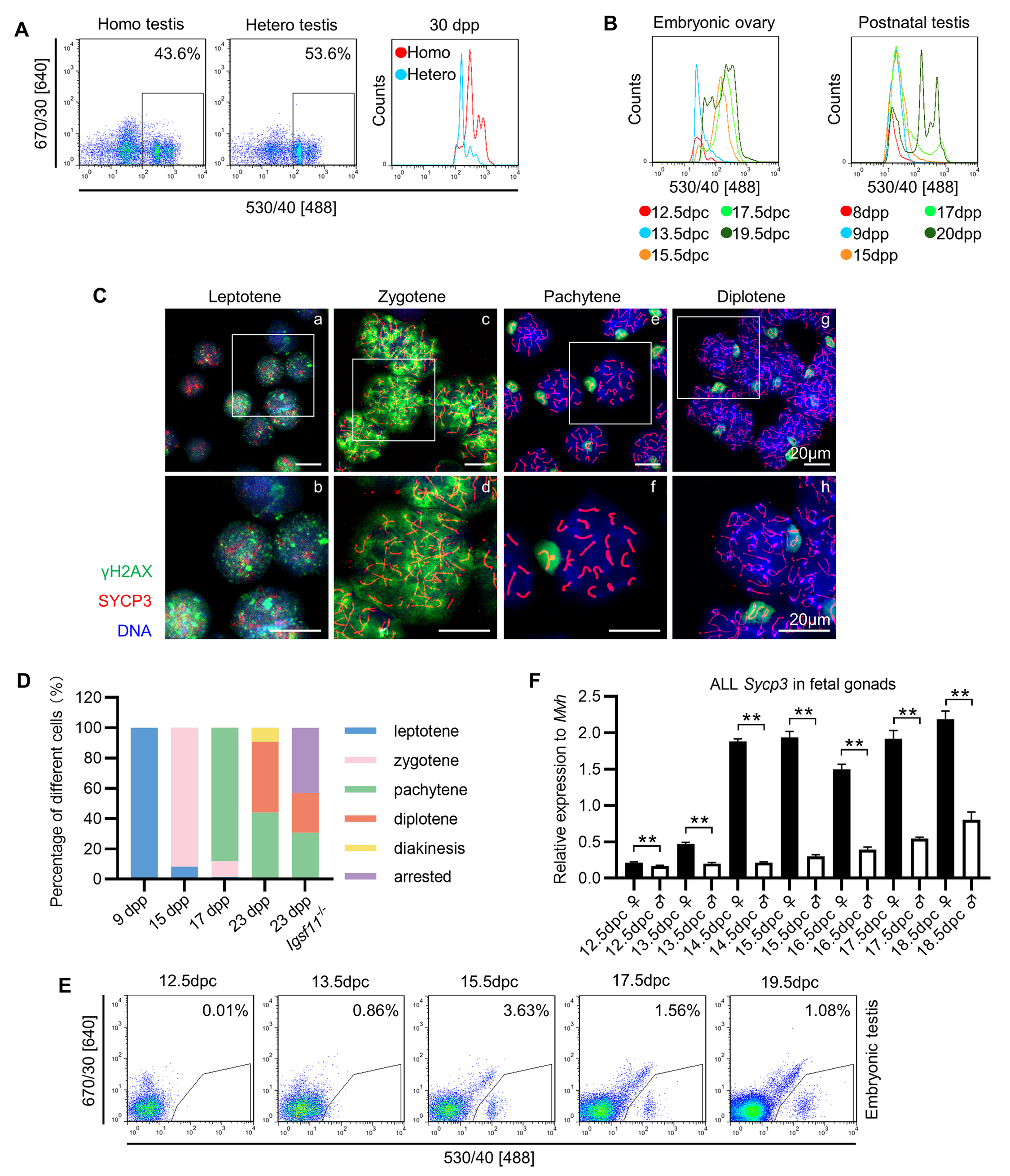

Supplement: S2 Fig — (A) Comparison of mVenus fluorescent intensity between 30dpp VPHS homozygote (Homo) and heterozygote (Hetero) littermates. (B) Comparison of mVenus fluorescent intensity in homozygote gonads between each developmental stage. Separated diagram for each developmental stage was shown in Fig 1E. (C-D) Meiotic spread staining analysis (C) and statistics (D) of spermatocytes sorted by the VPHS reporter from different developmental stages. Cells were sorted by the red gating shown in Fig 1E. n = 899 cells. (E) Detection of mVenus fluorescence within homozygotes fetal testis of the VPHS mice. (F) Expression of transgenic Sycp3 transcript within embryonic homozygote VPHS gonads. For each development stage, fetal gonads from two pregnant mice were pooled as one biological repeat for RNA extraction. Biological repeats = 1, Technical repeats = 3. Values and error bars are means and S.D. **, P < 0.01 by Student’s t test. (TIF) [file pgen.1009778.s002.tif]

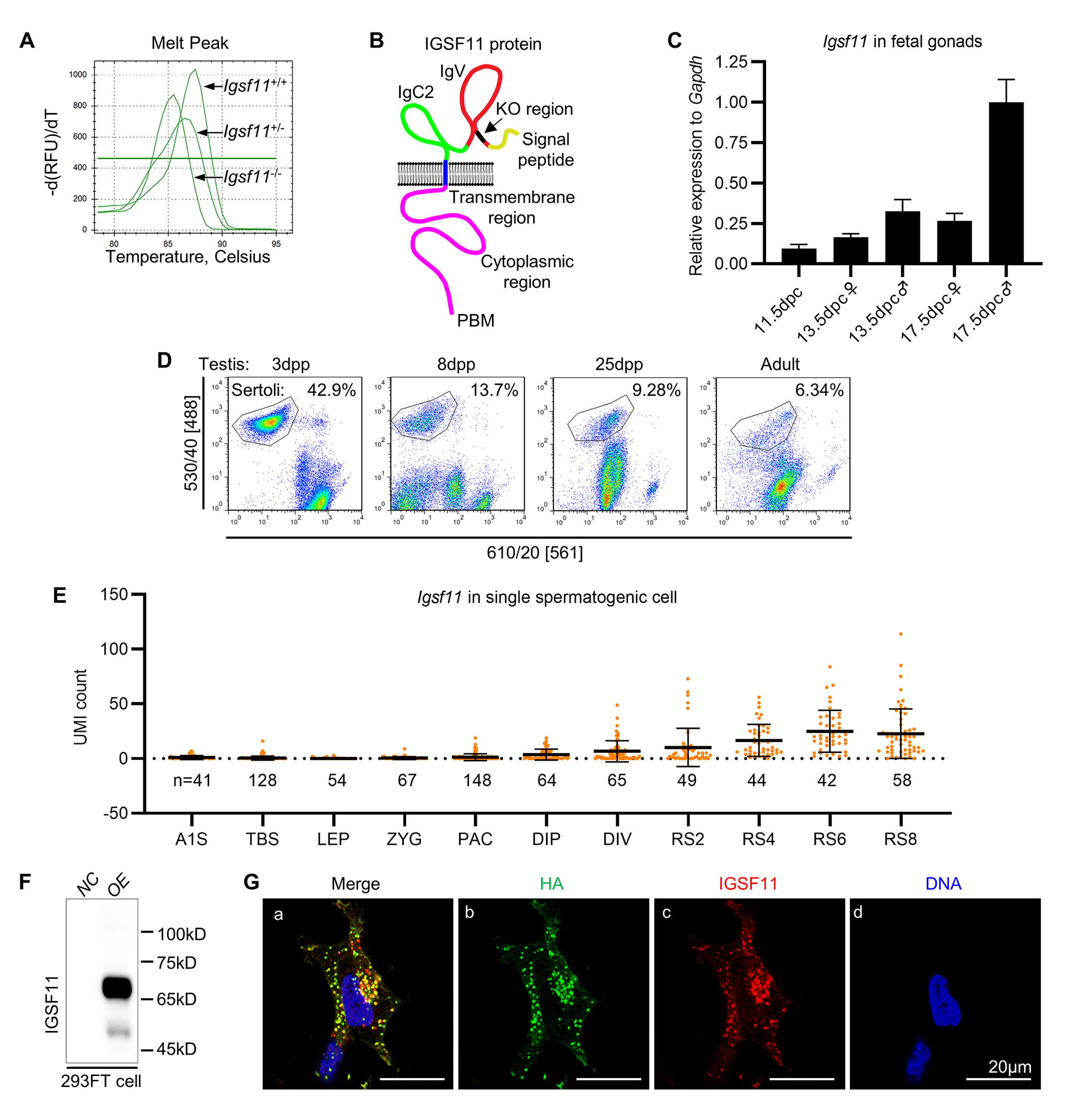

Supplement: S3 Fig — (A) Genotyping of Igsf11 knockout mice by melting curve of the Q-PCR product. (B) Illustration of IGSF11 as transmembrane protein. (C) Expression of Igsf11 in different stage of fetal gonad. Biological repeats = 1, Technical repeats = 2. Values and error bars are mean and SD. (D) Purification of mouse Sertoli cells by FACS. (E) Single cell expression profiling of Igsf11 in postnatal spermatogenic cells from previous study [20]. Abbreviations: A1S, type A1 spermatogonia; TBS, type B spermatogonia; LEP, leptotene spermatocyte; ZYG, zygotene spermatocyte, PAC, pachytene spermatocyte; DIP, diplotene spermatocyte; DIV, metaphase spermatocyte; RS2, steps 1–2 spermatids; RS4, steps 3–4 spermatids; RS6, steps 5–6 spermatids; RS8, steps 7–8 spermatids. n, number of spermatocytes. n = number of single cells. (F-G) Specificity of the anti-IGSF11 antibody was confirmed in human 293FT cells expressing HA-IGSF11 by Western blot (F) or immunostaining (G). n = 3 experiments. Abbreviations: NC, negative control; OE, overexpression IGSF11. (TIF) [file pgen.1009778.s003.tif]

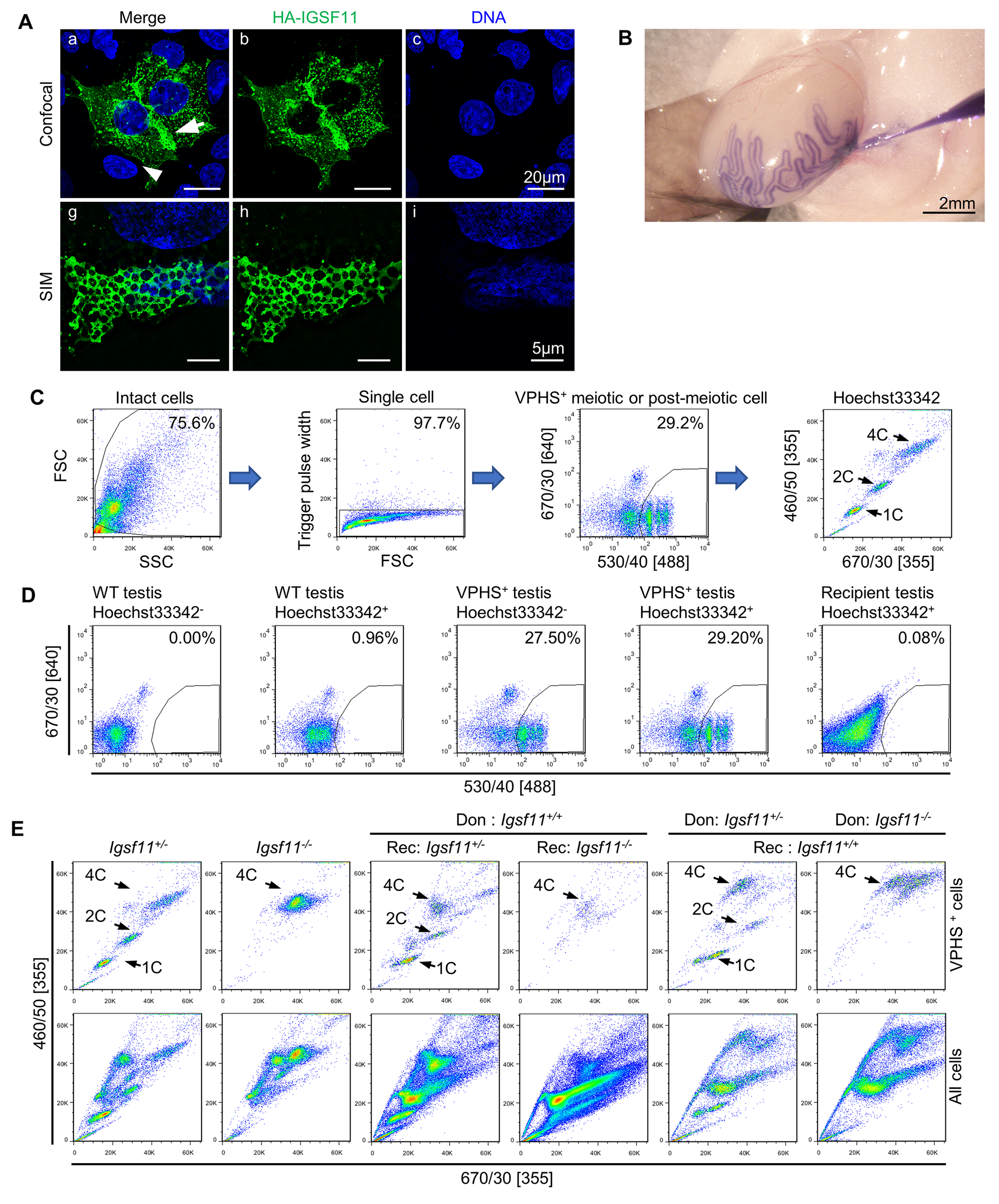

Supplement: S4 Fig — (A) Immunostaining of HA-IGSF11 in 293FT cells. Images were captured by confocal or super resolution SIM microscope. (B) Example of testicular cell transplantation surgery. Trypsinized testicular cells were resuspended in PBS containing Trypan Blue and injected into the recipient testis. Trypan Blue indicated donor cell-filled seminiferous tubules. (C) Gating strategy of V&H assay using VPHS transgenic testis. (D) Controls that help for the gating of VPHS+ cells. (D) Comparison between the results of V&H assay and traditional Hoechst33342 staining assay. (TIF) [file pgen.1009778.s004.tif]

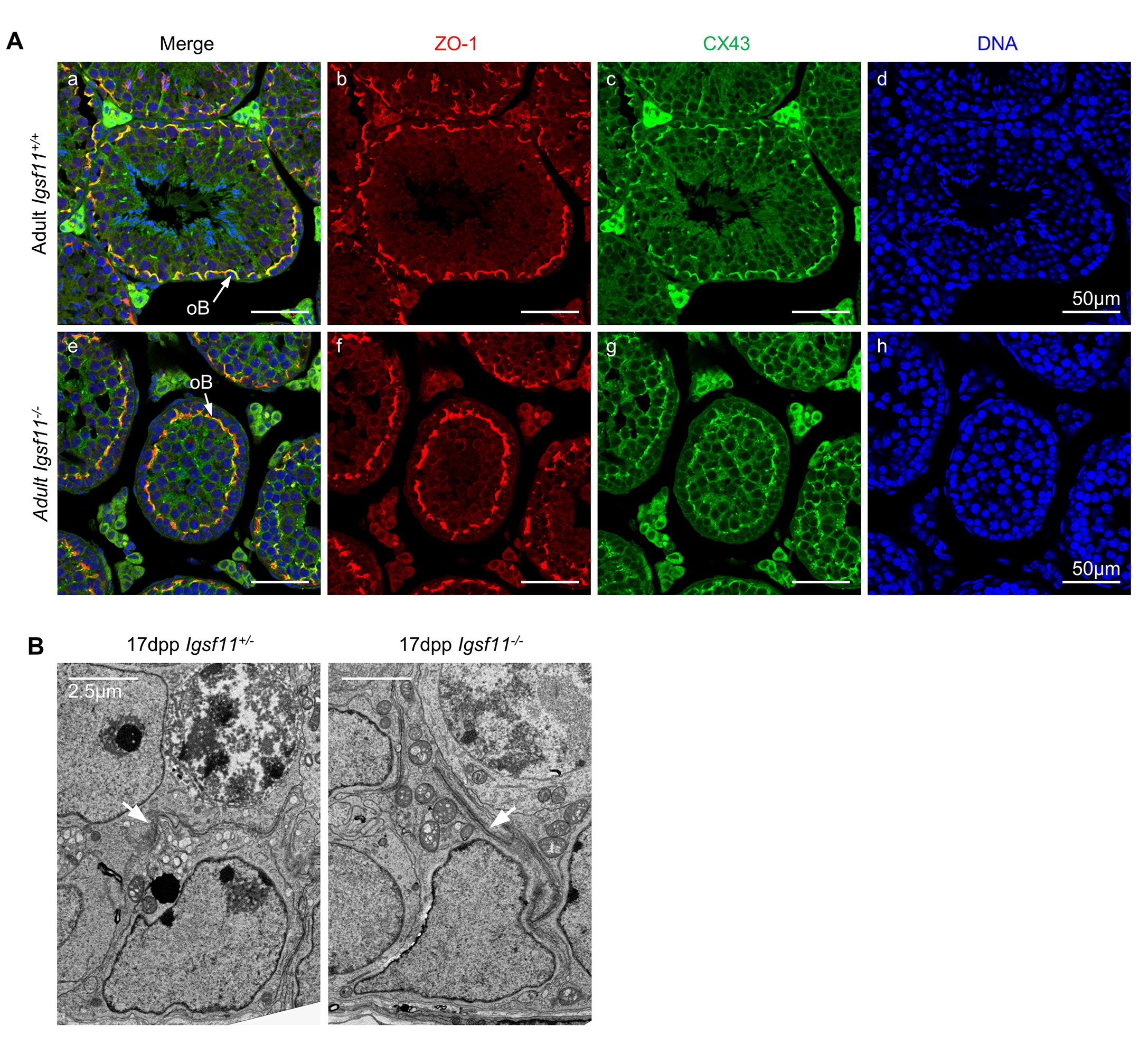

Supplement: S5 Fig — (A) Immunostaining of ZO-1 and CX43 in control and Igsf11 knockout adult testis. Abbreviations: ob, old BTB. (B) Transmission electron microscopy analysis of control and Igsf11 knockout 17dpp testis. BTB was indicated by arrow heads. (TIF) [file pgen.1009778.s005.tif]

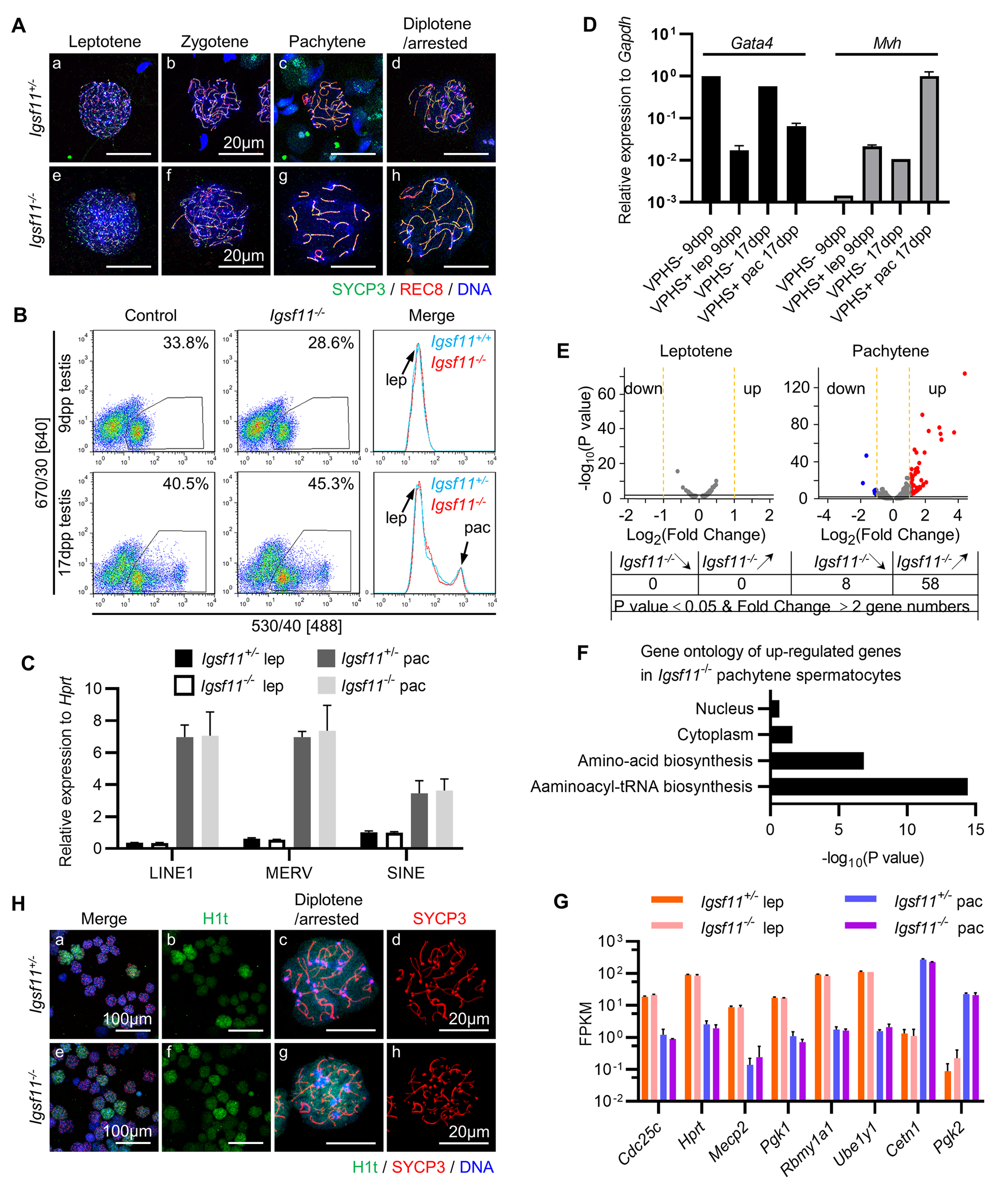

Supplement: S6 Fig — (A) Comparison of the axial element assembly in spermatocytes of the control (Igsf11+/-) and mutant (Igsf11-/-) mice using SYCP3 and REC8 as markers. (B) Meiotic progression analysis of the first wave spermatocytes with the VPHS transgenic reporter. (C) Q-PCR quantification of typical retrotransposon expression in purified leptotene and pachytene spermatocytes with different Igsf11 genotypes. Biological repeats = 3, Technical repeats = 3. (D-G) Transcriptome comparison of leptotene and pachytene spermatocytes between Igsf11 genotypes (E). Biological repeats = 2. Leptotene and pachytene spermatocytes were sorted with the help of the VPHS reporter (Fig 1E). Purity of the sorted cells were analysis by Q-PCR (D) and meiotic spread staining (S2C and S2D Fig). Gene ontology of up-regulated genes in Igsf11-/- pachytene spermatocytes (F). The expression of sex chromosome-linked genes (Cdc25c, Hprt, Mecp2, Pgk1, Rbmy1a1, Ube1y1) and compensatory genes on autosomes (Cetn1, Pgk2) that extracted from above mRNA-seq data (G). Abbreviations: lep, leptotene; pac, pachytene. (H) Incorporation analysis of H1t within meiotic prophase I spermatocytes. (TIF) [file pgen.1009778.s006.tif]

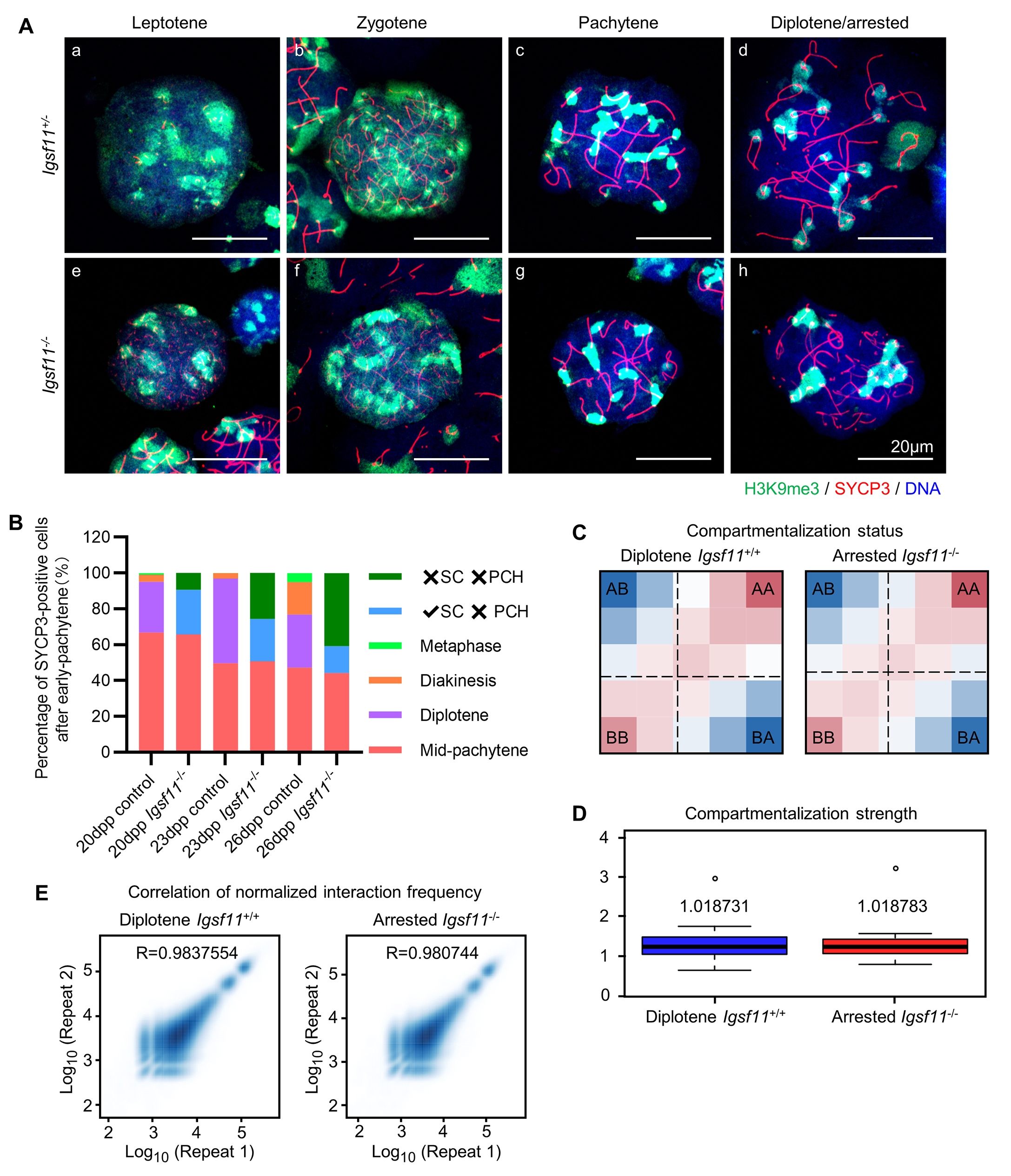

Supplement: S7 Fig — (A) Organization dynamic of PCH within primary spermatocytes of different Igsf11 genotypes. (B) Statistics of SYCP3-positive cells after early-pachytene with meiotic spread specimens. The normal or defective developmental status of the SC or PCH is indicated by "√" or "×", respectively. n = 868 spermatocytes. (C-E) Comparison of compartmentalization (C) and compartmentalization strength (D) between wild type and Igsf11 knockout spermatocytes. (E) Correlation of normalized interaction frequency among biological replicates in the Hi-C data. (TIF) [file pgen.1009778.s007.tif]
