# Supplementary material for: IGSF11 is required for pericentric heterochromatin dissociation during meiotic diplotene
Source: PLoS Genet. 2021 Sep 7;17(9):e1009778. doi: 10.1371/journal.pgen.1009778 (PMC8448346; doi:10.1371/journal.pgen.1009778)
Supplement: S1 Appendix — (DOCX) [file pgen.1009778.s015.docx]

**S1 Appendix**

**DNA sequence of recombinant *Sycp3* exon 1 in VPHS mice**

**Notes:**

The transgene was all integrated into the exon 1 of mouse *Sycp3*. Uppercase, lowercase, and red letters represent the transgene, the original exon, and the translation start site, respectively. The coding products of the transgene including mVenus fluorescent protein (238aa), GSG-P2A (22aa), HA tag (9aa) and an additional methionine.

**Sequence:**

atggcgttcagccaatcagcagagagcttggtcggggccggactgtatttactcctgcccaagggccaggtttcctcagatgGTGAGCAAGGGCGAGGAGCTGTTCACCGGGGTGGTGCCCATCCTGGTCGAGCTGGACGGCGACGTAAACGGCCACAAGTTCAGCGTGTCCGGCGAGGGCGAGGGCGATGCCACCTACGGCAAGCTGACCCTGAAGCTGATCTGCACCACCGGCAAGCTGCCCGTGCCCTGGCCCACCCTCGTGACCACCCTGGGCTACGGCCTGCAGTGCTTCGCCCGCTACCCCGACCACATGAAGCAGCACGACTTCTTCAAGTCCGCCATGCCCGAAGGCTACGTCCAGGAGCGCACCATCTTCTTCAAGGACGACGGCAACTACAAGACCCGCGCCGAGGTGAAGTTCGAGGGCGACACCCTGGTGAACCGCATCGAGCTGAAGGGCATCGACTTCAAGGAGGACGGCAACATCCTGGGGCACAAGCTGGAGTACAACTACAACAGCCACAACGTCTATATCACCGCCGACAAGCAGAAGAACGGCATCAAGGCCAACTTCAAGATCCGCCACAACATCGAGGACGGCGGCGTGCAGCTCGCCGACCACTACCAGCAGAACACCCCCATCGGCGACGGCCCCGTGCTGCTGCCCGACAACCACTACCTGAGCTACCAGTCCAAGCTGAGCAAAGACCCCAACGAGAAGCGCGATCACATGGTCCTGCTGGAGTTCGTGACCGCCGCCGGGATCACTCTCGGCATGGACGAGCTGTACAAGGGCAGCGGCGCCACCAACTTCAGCCTGCTGAAGCAGGCCGGCGACGTGGAGGAGAACCCCGGCCCCTACCCCTACGACGTGCCCGACTACGCCATGcttcgagggtgtggggacagcgacagctcaccggagccgctgag
