# Supplementary material for: IGSF11 is required for pericentric heterochromatin dissociation during meiotic diplotene
Source: PLoS Genet. 2021 Sep 7;17(9):e1009778. doi: 10.1371/journal.pgen.1009778 (PMC8448346; doi:10.1371/journal.pgen.1009778)
Supplement: Attachment — Submitted filename: Review of Chen et al_mckee.docx [file pgen.1009778.s016.docx]

Review of Chen et al. IGSF11 …. PLoSGen

This manuscript describes novel results indicating the possible existence of a signaling pathway between somatic Sertoli cells and spermatocytes dependent on the adhesion protein IGSF11 that is required (in both cell types) for meiotic progression beyond diplotene and for resolution of heterochromatic chromocenters beginning in late pachytene. Defects in a variety of processes are known to block meiotic progression in zygotene or pachytene but none that block progression in diplotene have been previously described. The experiments are thorough and well-controlled and the data support the conclusions. The cytological figures, especially the meiotic spreads are clear and beautiful. This study will open new territory for studying how heterochromatic packaging interfaces with meiotic progression, a topic that should be of broad interest. The manuscript is well-written overall but there is a general problem with figure legends that are too brief and uninterpretable graphs with undefined axes. There are also several specific problems, detailed below, that will require a minor revision.

Specific comments (tied to line numbers)

99-101: the description of the knock-in procedure is a bit confusing. The authors state that they inserted the tag immediately downstream of the start codon but also say that the guide RNAs targeted Cas9 cleavage to sites that are 89 and 51bp upstream of the transcription start site (abbreviated TSS). This seems odd because there would likely be a long stretch of nucleotides between the cleavage sites and insertion site. They say this both in the text and the legend to Fig. S1 but in the actual figure, S1A, the site marked TSS has an ATG which could be a start codon, an interpretation bolstered by the special color of the A. This leads me to think they actually targeted their cleavage sites 89 and 51 bp upstream of the start codon, which would make more sense. If this is the case, the authors just need to change their references in the text and legend to “translation start site” instead of transcription start site. However, if they really did mean transcription start site, they should state how far upstream of the insertion site the cleavage sites really are.

129-135: It’s not clear why the authors think the translation initiation site is different in the insertion allele than in WT. The authors cite the 4kD size difference between the wild-type (WT) and knock-in (KI) SYCP3 bands, but if the translation start site really is upstream of the normal site, that should affect the length of the Venus band, not the HA-SYCP band. Moreover, the length difference between the WT and KI SYCP3 proteins doesn’t seem remarkable. The SYCP3 protein in the knockin (KI) allele is expected to carry additional amino acids at its N- terminus: one proline derived from the P2A tag followed by however many amino acids are present in the HA tag. So the KI protein should be longer than the WT protein. How much longer depends on the copy number of HA in the tag, which the authors don’t state. A common HA tag has 3 copies and is 27 amino acids, corresponding to about 3.3kD. Adding the NT proline but subtracting the start methionine would leave the KI protein about 3.3kD larger than the WT protein, which is pretty close to the difference the authors actually detected in the Westerns (27 vs 31kD). The authors should specify the copy number of the HA peptide in their tag. And if they still believe that translation initiated at a different-than-normal site, they should explain their reasoning.

132-135 and Fig. 1C and Fig. S2C: the fact that the SYCP3 band is the same intensity in KI homozygotes as in WT is remarkable. P2A peptides usually cause a considerable reduction in expression level of the downstream protein (HA-SYCP here) because of ribosome dissociation and inefficient re-initiation.

Fig. 2 and Fig. S2: the plots in Figs. 1E, S2F & S2G need more explanation – what is APC and what is the X axis? How should readers interpret these plots?

189 & Fig. 2G – the authors should explain what Amh+ and Amh- refer to and explain what their significance is.

199: define BTB

Fig 2H legend should be more extensive. What are the various rows?

Fig 2I also needs a more thorough legend.

Fig. 3B: It’s not clear what the purpose of the second row of images (b, d, etc.) is. They are never mentioned in the text or figure legend. If readers are supposed to learn something from the images in 3B other than the intensity of the Venus signal, the authors need to explain what it is.

308-352: The authors do a pretty thorough job of comparing the mutant and wild-type spermatocytes on parameters related to pairing, recombination, gene expression and others. But there were no comparisons based on parameters related to cell death. It seems possible that the mutant spermatocytes are apoptotic or affected by another programmed cell death process, as is the case in spermatocytes with activated pachytene checkpoints.

383-386: This sentence contains a very cryptic reference to “SC abnormalities in the mutant nuclei”. What abnormalities? This is the only mention of them and no description or evidence is presented. I did wonder whether there might be a connection between the processes of SC disassembly and chromocenter dissociation as the two processes overlap temporally in late pachytene and diplotene. But that possibility was not explored. It seems there should be some way of characterizing and maybe quantifying the extent of SC disassembly in WT versus mutant. It would be interesting to know if there are correlated defects in these two important disassembly processes in late prophase I.

392-393 & 409-410: The authors claim that they purified diplotene stage spermatocytes from WT and mutant testes and that the Hi-C results that obtained reflect the situation that pertains at diplotene. But they do not offer any explanation of how they specifically isolated diplotene stage cells or any quality checks to evaluate the level of contamination with other cell types.

Discussion

The main findings are that IGSF11 is required both in Sertoli cells and spermatocytes for meiotic progression in male mice; that mutant spermatocytes arrest at diplotene; and that they fail to undergo disassembly of the chromocenters that cluster pericentric regions of non-homologs during pachytene. The two latter defects constitute an intriguing correlation. To their credit, the authors make no strong claims about a causal relationship, as there is no evidence for one, but they also never make the point that their result is nothing more than an association, which might be useful because readers that skim the paper might assume that one causes the other.

The suggestion that there might be a post-pachytene checkpoint that monitors events like chromocenter disassembly is an interesting one, albeit highly speculative. There are likely a number of alternative explanations that are not explored but a checkpoint seems one plausible mechanism and one that can be tested in a variety of ways. A possible weakness with this idea is that it doesn’t have any clear connection with the presumption that IGSF11 mutants impair signaling between somatic cells and spermatocytes implied by the cellular function of IGSF11. If the authors have any ideas about how the germ cell phenotypes might be related to possible soma-germ cell signaling (or germ cell-germ cell signaling), that could strengthen the discussion.
