# Supplementary material for: IGSF11 is required for pericentric heterochromatin dissociation during meiotic diplotene
Source: PLoS Genet. 2021 Sep 7;17(9):e1009778. doi: 10.1371/journal.pgen.1009778 (PMC8448346; doi:10.1371/journal.pgen.1009778)
Supplement: Attachment — Submitted filename: Reply to editors 20210809.docx [file pgen.1009778.s017.docx]

Dear reviewers,

We thank all reviewers for their recognitions and suggestions to our manuscript and study. These comments have strengthened our manuscript and help us clarifying points which are unclear to the readers. We have added more experimental data and manuscript editing to address reviewers’ comments.

Sincerely,

Bo Chen, Kehkooi Kee and all authors.

Responses to reviewer 1:

| Q  1 | In meiosis I, chromosomes exhibit a unique segregation pattern as homologous pairs segregate to different poles of the dividing cell. Studies such this on mechanisms that guarantee the proper segregation of chromosomes are important because errors in meiosis I are important causes of birth defects and mental retardation. Proper meiotic segregation patterns are achieved by meiosis-specific mechanisms such recombination, dissolution of the synaptonemal complex, and other aspects that alter chromosome structure and behavior such homologous and heterologous centromeric interactions. In this work by Chen et. al. the authors created a mouse model based on the expression of GFP under the control of the Sycp3 gene promoter and use this model for phenotypic analysis of IGSF11 knockout spermatocytes and oocytes. A remarkable aspect of this work is the generation and validation of Venus-P2A-HA-Sycp3. This alone may constitute a significative addition to the much-needed toolbox of mouse models and molecular strategies in the field. Except for relative minor observations (detailed next), the idea of a mice that can be used to easily isolate meiocytes at different stages of prophase I is exiting, and the data validating the model in this work is of excellent quality. Observations regarding this part of the work: Page 6. VPHS mice seems to have smaller testis. This together with a described effect of the described knock-in in specific variants expression of Sycp3, bring to my attention that this possible deficiency may have consequences in gamete development. Here, a careful characterization should determine differences of VPHS testis respect to control. This is by measuring testis weight, typifying seminiferous tubule types, and addressing apoptosis, among other possible analysis. In this endeavor, it may help clarifying what is known regarding the possible functions of a smaller Sycp3 variant. Although this should be addressed, the fact that DNA repair and early and mid-stages of meiosis are apparently normal is very encouraging. |
| --- | --- |
| A | Indeed, some relatively big denucleated structures were occasionally appeared in the homozygous VPHS tubules (S1B Fig arrowheads), and fewer sperms as well as more cell debris were presented in the homozygous epididymis than heterozygous or wild-type littermates (S1B Fig), suggesting a reduced spermatogenic activity in homozygous VPHS testis, which may explain the smaller testis size for VPHS homozygote. This information has been added to the text and figure. So far, we are not clear about when and why these cells were affected. However, since this is not a common phenomenon for all VPHS homozygous germ cells, we think that the application of the VPHS mice is not compromised if the appropriate experimental design and control is included (e.g., using VPHS mice of the same genotype between control and treatment groups). |

| Q  2 | In a second aspect, this manuscript describes the phenotype of IGSF11 deletion and claim that in absence of this protein cells arrest at diplotene like stage and non-homologous pericentromeric heterochromatin dissociation is affected. As presented the evidence may not support the claims and they need to strengthen the presentation of data.  1- Page 15, starting in line 309. “Spermatocytes cannot develop beyond diplotene”. Evaluation of figures leads me to the conclusion that abnormal diplotene may actually represent apoptotic pachytene-like cells? In this case, the manuscript will need adjustments in the interpretation of the data.  In the same stream, it is possible that the process of exit from pachytene to be affected, accounting for other described phenotypes. A possible way to address this is by evaluating the state of chaperones such as HSP70 and partners. Related to this, in Figure 6 and page 18, again, are the “diplotene” cells actually in diplotene stage, or they are apoptotic cells? So actually, that results may be explained by cells being arrested in pachytene and accumulating defects as they become apoptotic?  Also, I think the focus of attention should be late pachytene and early diplotene (please quantitate), where axis decomposition has not yet occurred. |
| --- | --- |
| A | In the original manuscript, we concluded that the mutant cells entered diplotene stage based on several evidence, including SYCP1 staining pattern showing partially dissociated homologous chromosomes, which might not be obvious to the reviewer. In the updated figure, we have added more confocal images of diplotene stage *Igsf11* mutant images to Fig 5B. In these cells, SYCP1 were gradually disappearing from the SC where the lateral element dissociated from each other, while the dissociation of nonhomologous PCH was arrested. Based on these results, we concluded that *Igsf11* deficient spermatocytes indeed entered diplotene.  Another evidence supporting that the mutant cells have entered diplotene is the staining of H1t. H1t is a common mid/late pachytene marker for spermatocytes. Our immunostaining result (S6H Fig) and quantitative result (Fig 5E) showed that H1t was elevated to a comparable level between the diplotene like mutant spermatocytes and the control cells, suggesting the mutant cells developed beyond pachytene.  Regarding apoptotic stage of the mutant cells, we have conducted TUNEL assays on both testis and epididymis and found that more TUNEL positive cells are in epididymis. The TUNEL stainings of testis showed that a few apoptotic cells were closed to the basal laminar of both control and mutant seminiferous tubules (Fig 5F a b). Based on their cellular locations close the basal laminar, these apoptotic cells which appear in both wild-type and mutant, were not likely to be meiotic cells that were undergoing pachytene. Moreover, TUNEL signal was not observed in the cells which located in the middle part (more likely to be pachytene or diplotene cells) of the mutant seminiferous tubules (Fig 5F b). TUNEL assay with meiotic spread specimen reconfirmed that *Igsf11* mutant spermatocytes were lack of TUNEL signal when their SC became fragmented (Fig 5G a b). Remarkably, apoptotic cells with strong TUNEL signal intensity were observed in the mutant epididymis but not in wild-type epididymis, and some TUNEL-negative cells with distinct chromocenters were also observed (Fig 5F d). Hence, we concluded that the mutant cells were most likely undergoing apoptosis after they are released into epididymis at diplotene-arrested cells. |

| Q  3 | 2- It is possible that failures observed in IGSF11 knockouts could be ascribed to changes in chromosome associations to the nuclear membrane (perhaps related to the LINC complex ?) This could explain changes in pericentromeric chromatin clusters and relative later prophase I phenotype (i.e., increased chromosome entanglements?). This view is supported by IGSF11 being a membrane protein. In principle, this could be assessed by evaluation of telomere and LINC proteins localization in spermatocyte squashes. |
| --- | --- |
| A | We have tested this hypothesis, but the localization of SUN1 and TERF1 was not affected in *Igsf11*^-/-^ spermatocytes. |

| Q  4 | 1- The work fails to describe previous relevant work on pericentromeric chromatin association dynamics in mice (e.g., work from the Dawson and Sherthan labs ). A careful comparative analysis will be useful for readers and to determine significance of this work. |
| --- | --- |
| A | We have cited a relevant work from Dean Dawson’s lab in our original text. The sentence is “Within mouse spermatocytes, PCH undergoes dynamic assembly and dissociation during meiotic prophase I, and more importantly, the dynamics of PCH is highly correlated with that of the SC (Figs 6A and S7A; and [37]), suggesting that the dynamics of PCH is also regulated and coordinated by the meiotic program.”  In our revised text, Harry Scherthan’s work has been cited in the same sentence. |

| Q  5 | 2- Excess of acronyms are complicating the reading, for example HT and HO . |
| --- | --- |
| A | These abbreviations were replaced with Hetero and Homo, respectively, both in the text and figures. |

| Q  6 | 3- Figure 1E. Indicate % corresponding to Leptotene and zygotene in 20 dpp and if possible, in Figure S2G. |
| --- | --- |
| A | Although there are clusters of cell population with different intensities in 20 dpp (Fig 1E), the purity of leptotene and zygotene meiocytes in 20dpp are lower than 9 or 15 dpp due to the mixed populations in later developmental time-points, so we did not label the clusters. Based on our results, isolation of spermatocytes during the first wave and at the earliest developmental time-points will yield the highest purity. |

| Q  7 | 4- Methods and Materials description is insufficient. Please, revise the entire Method section with special attention to a- RNA-seq methods and analysis of results, and 2-obtention and validation of enriched fractions of meiotic and not meiotic cell populations. Validation of the enriched cell fractions is very important to assess the accuracy of results interpretation. |
| --- | --- |
| A | We have revised the methods part, and validation data have been added to S2D and S6D Figs. |

| Q  8 | 5- Quantitation and reproducibility:  a- In all experiments is not immediately clear number of mice used and biological replicates. Please revise and add in the text or figure legend. In the same line, please review and reach minimum statistical number (or at least justify current data validation) for transplant experiments and cell sorting experiments.  b- Quantitation (or mentioning the number of repetitions) may be required in several figures and experiments. For example, Fig 1F, Fig 2H, Fig 4, Fig 5, and FigS5B, part of Fig 6. |
| --- | --- |
| A | We have revised this issue by adding duplicate information of every quantitative data to the figure legend. Every FACS result was repeated for at least three times with three biological duplicates, and was stated in the method section. |

| Q  9 | c- RNA seq experiments are inadequately described leaving critical information regarding procedure and statistics (e.g., repetition) incomplete. Equally important, the description of results in the text is not sufficient. As presented data do not support conclusions in page 17 line, 347-352. Careful analysis of gene sets for x-y inactivation, retrotransposon silencing, and genome wide transcription changes (particularly in genes that may affect synapsis, recombination repair and other essential meiotic process) need to be better assessed and documented. Why do they use sex chromosome linked genes? Is this to assess X-Y silencing? Are genes involved in pachytene exit affected (these are not part of the pachytene checkpoint). Regarding the material used, what purified leptotene and pachytene means?  d- They should also consider that even if transcription is not affected, localization of critical factor may, thus causing phenotype. |
| --- | --- |
| A | We have clarified the procedures and details of the RNA-seq in the method section. Besides RNA-seq, Q-PCR (and western blot) screening of many known meiotic related genes were also performed (data not shown in text). These results helping us to exclude the possibility of IGSF11 function in transcription regulation of meiotic genes.  We also have revised corresponding text in the manuscript to make it clearer. |

| Q  10 | e- Quantitation of experiments appear in supplementary data, please integrate this data into the main figure (example, testis size quantitation and data in FigS6A).  f- please provide entire data sets. |
| --- | --- |
| A | We have revised this issue. |

| Q  11 | 6- Page 13, line 276. Replace significantly for apparently. |
| --- | --- |
| A | We have corrected this description. |

| Q  12 | 7- Fig 4B – page 14, line 290. What criteria was used to classify Pre-leptotene cells. This is not immediately clear as no marker was used. |
| --- | --- |
| A | We are actually indicating the cells which have only a weak γH2AX staining, and after reading your reminder, we think it is more appropriate to use “early-leptotene” to define the stage of these cells.  We have revised this description in both text and figures. |

| Q  13 | 8- In figures and text (example pag15, line 316). What abnormal diplotene means? What is the criteria to classify these cells as diplotene? As indicated above, are they diplotene or cells arrested in pachytene and undergoing apoptosis? |
| --- | --- |
| A | These questions are similar to question 2, which have been replied. |

Responses to reviewer 2:

| Q  14 | How germ cells and gonadal somatic cells work together to regulate meiosis is unclear. In this manuscript, the authors constructed a mVenus-P2A-HA-Sycp3 reporter mice which can be used to examine the progression of meiotic prophase I and isolate meiotic cells from mouse testes. Then, the authors identified an adhesion molecule IGSF11, which is expressed in both sertoli cells and germ cells. With the help of the new reporter mice and testicular cell transplantation, they demonstrated that IGSF11 expressed in both sertoli and germ cells is required for meiosis completion in spermatocytes. The authors further showed that in the absence of IGSF11, spermatocytes proceed through pachytene without obvious defects, but the pericentric heterochromatin of nonhomologous chromosomes could not dissociate properly. The abnormal interchromosomal interactions was further confirmed by Hi-C analysis. This study suggests IGSF11 plays an important role in regulating the coordination between sertoli and germ cells for proper meiosis progression. Experiments are well done and convincing. I only have several minor concerns.  1. Page6 line 126, please specify "corresponding expression levels". |
| --- | --- |
| A | Original sentence: HA-SYCP3 and mVenus were only detected with corresponding expression levels in the heterozygous and homozygous VPHS samples.  Corrected sentence: HA-SYCP3 and mVenus were only detected in the heterozygous and homozygous VPHS samples, and their expression in homozygous is higher than that in heterozygous. |

| Q  15 | 2. p7 lies130-135, does the longer isoform have any special functions? |
| --- | --- |
| A | The shorter SYCP3 isoform is conserved among vertebrates, and the longer one is formed in the common ancestor of rat and mouse (Alsheimer et al., 2010). The functional difference between these two isoforms has not been discussed in the literature so far. |

| Q  16 | 3. P7, it would be better to show the reporter mice have normal fractions of spermatocytes in different substages (leptotene, zygotene, etc). |
| --- | --- |
| A | As shown in S1B Fig, despite some abnormal cell occasionally found in the VPHS homozygote, the overall histological structural in similar between different VPHS genotypes. Moreover, in S1I Fig, 17 dpp testes with three different VPHS genotypes contained similar number of pachytene spermatocytes in the cross section of their seminiferous tubule; in S2A Fig, the number of 1C population (spermatid) is comparable between VPHS homozygote and heterozygote. Based on these results, we assumed that the reporter mice have normal fractions of spermatocytes in different substages. |

| Q  17 | 4. P10 line 203, authors should note that SYCP3 is an axis component. |
| --- | --- |
| A | We’ve mentioned this information in the section about the VPHS mice. Regarding sentence is “These data suggest that transgene insertion did not influence the meiotic-specific expression pattern, the localization to chromosome axes and the synaptonemal complex (SC), or the degradation after meiotic prophase of HA-SYCP3”.  In the context of this sentence, it might more appropriate to describe SYCP3 as a primary spermatocyte marker protein.  So the original sentence “Coimmunostaining of IGSF11 with Sertoli cell marker GATA4 or the SC marker SYCP3 further confirmed IGSF11 expressed in the cytoplasm of both cell types” was revised to “Coimmunostaining of IGSF11 with Sertoli cell marker GATA4 or primary spermatocyte marker SYCP3 further confirmed IGSF11 expressed in the cytoplasm of both cell types”. |

| Q  18 | 5. Figure 1 and the legends: I did not see gating parameters in Figure 1E.  Is it possible to do the same analysis as in panel F to confirm the sorted meiotic cells in female? |
| --- | --- |
| A | We have added the gating parameters to Figure 1E. Yes, it is possible to do the same analysis for female germ cells but need a more detailed and comprehensive study using this reporter mice for female germ cells. We are preparing these datasets for a future study at this moment. |

| Q  19 | 6. Figure 2C, IGSF11 have two isoforms? If so, please clarify, if not, please explain why there were two bands. |
| --- | --- |
| A | Based on NCBI database, IGSF11 has only one transcription or translation isoform in mice. These two bands might be two different post-translational modified IGSF11 migrating to different location in SDS-PAGE. We have added this speculation to the revised figure legend. |

| Q  20 | 7. Figure 2FG, please specify the error bar and how many times experiments were repeated. |
| --- | --- |
| A | We have added the information. |

Responses to reviewer 3:

| Q  21 | In this interesting manuscript the authors report on the male infertility phenotype of deficiency of the immunoglobulin-like cell adhesion molecule IGSF11, significantly and informatively adding to a previous study. They document IGFS11 expression in both Sertoli cells and germ cells and show that targeted mutation of the Igfs11 gene leads to infertility with arrest of spermatocytes at a late pachytene stage, prior to diplotene. Germ-cell transplantation analyses revealed that for normal gametogenesis, IGSF11 is required in both Sertoli cells and germ cells. The authors show abnormal clustering of the pericentric heterochromatin (PCH) of nonhomologous chromosomes at late pachytene, shown also by Hi-C analysis, resulting in failure of chromosome individualization. Another nice feature of this manuscript, albeit somewhat unrelated to the main theme, is the development of a mVenus SYCP3 reporter system (VPHS) that facilitated analysis of the progress of meiosis in spermatocytes as well as cellular localization in testis tubules.  Overall, this is a great scientific study, presenting new information, and commendable for the depth of analysis and incredible thoroughness in attention to detail. The work is competently performed, with appropriate controls, and the figures are, for the most part, excellent, with only a few suggestions for improvement.  The transplantation experiments are especially appreciated. However, this should have a bit more explanatory rationale for readers unfamiliar with the technique. |
| --- | --- |
| A | We have described more details and information about the transplantation experiments to in the text before describing the results of Fig 3. |

| Q  22 | The major concern about this manuscript is with respect to the striking similarity of the *Igsf11* KO germ-cell phenotype to the germ-cell phenotype in mice lacking androgen receptor in Sertoli cells (the SCARKO mutant model). Clearly this study has provided more information about the abnormal diplotene and compromised dissociation of the PCH, which has not been explicitly investigated in SCARKO mice. However, it is difficult to imagine a direct mechanism by which IGSF11 would control PCH dissociation, rather than it being a consequence of arrest of meiotic progression. Thus, it would be interesting for the authors should comment on the similarities and differences of *Igsf11* KO and SCARKO phenotypes; this consideration could yield insights into possible mechanisms of IGSF11 action (could it be downstream of AR, and facilitate androgen signaling to the germ cells?). It would also be informative to comment on the apparent lack of differences in the genome-wide transcription profile of the *Igsf11* mutants in the context to what is known about gene expression in SCARKO mutants. |
| --- | --- |
| A | After comparing phenotypes of Igsf11 KO to the published phenotypes of SCARKO, we think there are phenotypic and mechanistic differences between Igsf11 KO and SCARKO mice: 1. Transcriptome of SCARKO spermatocytes is arrested at leptotene/zygotene to early-midpachytene transition, but Igsf11 deficient spermatocytes do not (Larose et al., 2020).  2. SCARKO spermatocytes accumulate many undissolved γH2AX signals but Igsf11 deficient spermatocytes do not (Chen et al., 2016; Larose *et al.*, 2020).  3. SCARKO spermatocytes undergo apoptosis within seminiferous tubules, but Igsf11 mutants undergo apoptosis in epididymis (De Gendt et al., 2004).Hence, these differences indicated that SCARKO arrested at early phase (leptotene/zygotene) of meiotic prophase but Igsf11 KO arrested at the later phase (diplotene). |

| Q  23 | The transgenic VPHS reporter will be a useful resource to others; information on how it will be made available would be helpful. |
| --- | --- |
| A | We are happy to distribute and share the transgenic mice with the research community. At this moment, we had already sent these mice to other researchers interested in studying meiosis. Please contact us directly by email. |

| Q  24 | 2. The authors should take care not to confuse DNA content, C, with chromosome content, N. For example, spermatocytes are 4C (post S-phase), but 2C (and definitely not “tetraploid,” as often erroneously stated in the literature!). This must be corrected throughout the text and figures. |
| --- | --- |
| A | We have revised this mistake. |

| Q  25 | 3. Fig. 2H needs better legend and labels to point out features discussed in text, as well as clearer validation of the specificity of the antibody. As presented, it appears that staining is not robust (Fig. 2l-n) or histology is unclear (Fig. 2o-r). |
| --- | --- |
| A | The specificity of the IGSF11 antibody has been verified by WB (S3F Fig) and immunostaining (S3G Fig). Comparing to the staining result from *Igsf11*^-/-^ testis as negative control (Fig 2H s-v), the dot signals (Fig 2H a-r) are believed to be IGSF11 containing secretory vesicles. In these experiments, we used the squash method to prepare staining samples, histology is less preserved with this method than sections. |

| Q  26 | 4. Fig. 4B (“ob” and “nb”) is difficult to interpret and could be improved with either (or both) higher magnification or a diagram. Alternatively, Fig. S5B could be pulled into the main text as more convincingly illustrative of the BTB. |
| --- | --- |
| A | We have added a diagram to illustrate the staining results. |

Responses to reviewer 4:

| Q  27 | The authors developed an ingenious meiotic reporter based on the knock-in insertion of a fluorescent protein in the meiotic gene Sycp3. They combined this reporter with an pre-existing Hoechst staining-based sorting protocol in order to sort meiotic germ cells at different stages among testes cells with increased specificity. They then generated a KO mouse model for IGSF11, an adhesion protein expressed both in Sertoli cells and in germ cells within the testis. IGSF11 is required for completing male meiotic prophase, and the authors show by an elegant testis cell transplantation assay that IGSF11 expression is required both in Sertoli cells and in germ cells. They show that spermatogenesis is arrested during the diplotene stage of meiotic prophase I, representing a new arrest point, and that it is characterized by a defect in chromosome reorganization within the nucleus in the late prophase. Although this report does not permit to understand the mechanism by which the lack of the adhesion protein IGSF11 leads to this defect, there is no doubt that it provides an insightful description of the spermatogenesis defects of this mutant that will be a reference for further studies (in addition to describing the generation of a novel, useful reporter). The study is conducted well, the experiments are well done and interpreted carefully, and the manuscript is clear and well written. I have only few concerns listed below.  Pages 15-17: The abnormal diplotene stage shown on figures 5 and S6 is not described in the main text. Early, middle and late-diplotene are shown on figure 6A, but it is not explained how these substages are defined in the mutant, where SYCP3-defined axes appear to become fragmented faster than in WT. What is the proportion of each sub-stage compared to WT? |
| --- | --- |
| A | We have added more description on how we defined diplotene and decided the arrested diplotene for Figure 5 and 6. In the original manuscript, we concluded that the mutant cells entered diplotene stage based on several evidence, including SYCP1 staining pattern showing partially dissociated homologous chromosomes. In the updated figures, we have added more confocal images of diplotene stage *Igsf11* mutant images to Fig 5B. In these cells, SYCP1 were gradually disappearing from the SC where the lateral element dissociated from each other, while the dissociation of nonhomologous PCH was arrested. Based on these results, we concluded that *Igsf11* deficient spermatocytes indeed entered diplotene.  It is difficult to determine that the developmental substage of control and mutant spermatocytes are exactly the same when the SC complex was fragmented. So, we do not know whether SYCP3-defined axes appear to become fragmented faster in the mutant than in WT. |

| Q  28 | This would give information about whether the spermatocytes are arrested and/or eliminated throughout diplotene stage or at the end of it. A more detailed analysis of histological sections would allow to define more precisely the stage of spermatocyte apoptosis (e.g. stage XII, or earlier). |
| --- | --- |
| A | Our TUNEL assay result revealed that the arrested diplotene cells in the mutant mice were released into the epididymis, where they underwent apoptosis (Fig 5F and 5G). |

| Q  29 | Fig 6E and 7: HiC data shown on figure 7D indicate a relative increase of end-end interchromosomal interactions, including proximal-distal interactions. This is reminiscent of the bouquet stage, which occurs normally earlier and is characterized by a reduced number of PCH clusters, too. This might be examined with an additional centromere-specific marker, which would allow distinguishing proximal from distal telomeres on spermatocyte spreads. It is indeed not clear whether only proximal telomeres associate with the clusters of PCH, nor whether every proximal telomere does so. |
| --- | --- |
| A | We co-stained ACA (anti-centromere protein antibody) with SYCP3, showing that autosome PCH were clustered to form several big chromocenters in *Igsf11* mutant spermatocytes, while their sex chromosome PCH were isolated (Fig 6D). |

| Q  30 | Discussion: A disfunction of BTB was suggested as a possible main cause for infertility in ref 8. The authors propose here a maybe more subtle defect because of the normal-looking BTB. This discrepancy should be discussed. More generally, whereas the germ cell arrest is often reported to occur much earlier than diplotene stage in mutants disrupting BTB function, this is not always clear and should be discussed. While I’m not a specialist, I’m not sure that the histological examination (markers and EM) is sufficient to conclude about the integrity of the BTB without a functional assay (diffusion of some compound such as shown in Xu et al., 2009, Mol Biol Cell 20:4268). |
| --- | --- |
| A | Based on the newly added TUNEL results and more detailed characterizations of substages in which Igsf11 mutant is arrested, we confirmed that the arrested diplotene meiocytes did not undergo apoptosis until they are in epididymis. Hence, this supports our conclusion that BTB defect is subtle and not detrimental to the early prophase meiocytes. Nevertheless, our assessment of BTB integrity may not be as thorough without other functional assays such as the one suggested by the reviewer and we thanked the reviewer suggestion. Because of the subtle phenotype and lack of more evidence to rule out the phenotype reported by other group, we did not discuss more about BTB in the discussion. We hope that the reviewer could understand our position. |

Responses to reviewer 5:

| Q  31 | This manuscript describes novel results indicating the possible existence of a signaling pathway between somatic Sertoli cells and spermatocytes dependent on the adhesion protein IGSF11 that is required (in both cell types) for meiotic progression beyond diplotene and for resolution of heterochromatic chromocenters beginning in late pachytene. Defects in a variety of processes are known to block meiotic progression in zygotene or pachytene but none that block progression in diplotene have been previously described. The experiments are thorough and well-controlled and the data support the conclusions. The cytological figures, especially the meiotic spreads are clear and beautiful. This study will open new territory for studying how heterochromatic packaging interfaces with meiotic progression, a topic that should be of broad interest. The manuscript is well-written overall but there is a general problem with figure legends that are too brief and uninterpretable graphs with undefined axes. There are also several specific problems, detailed below, that will require a minor revision.  Specific comments (tied to line numbers)  99-101: the description of the knock-in procedure is a bit confusing. The authors state that they inserted the tag immediately downstream of the start codon but also say that the guide RNAs targeted Cas9 cleavage to sites that are 89 and 51bp upstream of the transcription start site (abbreviated TSS). This seems odd because there would likely be a long stretch of nucleotides between the cleavage sites and insertion site. They say this both in the text and the legend to Fig. S1 but in the actual figure, S1A, the site marked TSS has an ATG which could be a start codon, an interpretation bolstered by the special color of the A. This leads me to think they actually targeted their cleavage sites 89 and 51 bp upstream of the start codon, which would make more sense. If this is the case, the authors just need to change their references in the text and legend to “translation start site” instead of transcription start site. However, if they really did mean transcription start site, they should state how far upstream of the insertion site the cleavage sites really are. |
| --- | --- |
| A | Mouse *Sycp3* gene has only one transcription isoform, which indeed start with “atg” nucleotides but have no translation start activity.  The induced cleavage site is -168b and -130b upstream *Sycp3* translation start site. We have revised our description in the text. |

| Q  32 | 129-135: It’s not clear why the authors think the translation initiation site is different in the insertion allele than in WT. The authors cite the 4kD size difference between the wild-type (WT) and knock-in (KI) SYCP3 bands, but if the translation start site really is upstream of the normal site, that should affect the length of the Venus band, not the HA-SYCP band. Moreover, the length difference between the WT and KI SYCP3 proteins doesn’t seem remarkable. The SYCP3 protein in the knockin (KI) allele is expected to carry additional amino acids at its N- terminus: one proline derived from the P2A tag followed by however many amino acids are present in the HA tag. So the KI protein should be longer than the WT protein. How much longer depends on the copy number of HA in the tag, which the authors don’t state. A common HA tag has 3 copies and is 27 amino acids, corresponding to about 3.3kD. Adding the NT proline but subtracting the start methionine would leave the KI protein about 3.3kD larger than the WT protein, which is pretty close to the difference the authors actually detected in the Westerns (27 vs 31kD). The authors should specify the copy number of the HA peptide in their tag. And if they still believe that translation initiated at a different-than-normal site, they should explain their reasoning.  132-135 and Fig. 1C and Fig. S2C: the fact that the SYCP3 band is the same intensity in KI homozygotes as in WT is remarkable. P2A peptides usually cause a considerable reduction in expression level of the downstream protein (HA-SYCP here) because of ribosome dissociation and inefficient re-initiation. |
| --- | --- |
| A | Mouse *Sycp3* gene has only one transcription isoform but 2 translation isoforms, so there are two SYCP3 bands (27.2kD and 29.3kD) in the wild type testis samples. The shorter isoform is 20aa (MLRGCGDSDSSPEPLSKHLK, which predicted to be 2.1kD) shorter than the larger one and has a higher translation efficiency (Alsheimer *et al.*, 2010).  Our transgene was integrated right after the upstream translation start site of *Sycp3* and contain only one copy of HA, so after cleavage of P2A, the transgenic HA-SYCP3 is 10aa (PYPYDVPDYA, which predicted to be 1.5kD) larger than the wild-type larger SYCP3 isoform.  So, if both translation start site were used in the transgenic transcript, there will be a 30.5kD HA tagged larger SYCP3 isoform and a 27.2kD wild-type shorter SYCP3 isoform. While compared to the wild-type testis, the shorter SYCP3 isoform was totally absent from the VPHS homozygote, and only 30.5kD HA tagged larger SYCP3 isoform was detected. The above information has been added to the revised text. |

| Q  33 | Fig. 2 and Fig. S2: the plots in Figs. 1E, S2F & S2G need more explanation – what is APC and what is the X axis? How should readers interpret these plots? |
| --- | --- |
| A | We have further standardized the format for indicating the axes of FACS data in the figures by displaying the parameters of the detection channel, and the information about these detection channel has been added when they first appeared in Fig 1E. |

| Q  34 | 189 & Fig. 2G – the authors should explain what Amh+ and Amh- refer to and explain what their significance is. |
| --- | --- |
| A | We used AMH+ to refer to the Sertoli cells in the original manuscript, but it is better to directly label the cells as Sertoli or non-Sertoli cells. We have replaced Amh+ or Amh- to Sertoli or non-Sertoli in the corresponding figures and legend. |

| Q  35 | 199: define BTB  Fig 2H legend should be more extensive. What are the various rows?  Fig 2I also needs a more thorough legend. |
| --- | --- |
| A | We have added the information to the legends. |

| Q  36 | Fig. 3B: It’s not clear what the purpose of the second row of images (b, d, etc.) is. They are never mentioned in the text or figure legend. If readers are supposed to learn something from the images in 3B other than the intensity of the Venus signal, the authors need to explain what it is. |
| --- | --- |
| A | Basically, the bright field channel pictures were used to show the presence of testis, as the mVenus signal from *Igsf11*^-/-^ spermatocytes is very weak (Fig 3B c g m).  Furthermore, the testis size is also informative as it is correlated with the spermatogenic activity of the testis. Since donor cells generally do not fully restore the spermatogenic capacity of recipient testes in transplantation experiments. So, recipient testis transplanted with donor cells should always larger than the non-transplanted recipient testis but smaller than the non-germ cell depleted testis. In our experiment, the observed testis size of different groups follow the expected rule: b＞f/k; d＞h/n. As we didn’t provide testis size measurement data, we decided not to mention this layer of information in the text but present the bright field images. |

| Q  37 | 308-352: The authors do a pretty thorough job of comparing the mutant and wild-type spermatocytes on parameters related to pairing, recombination, gene expression and others. But there were no comparisons based on parameters related to cell death. It seems possible that the mutant spermatocytes are apoptotic or affected by another programmed cell death process, as is the case in spermatocytes with activated pachytene checkpoints. |
| --- | --- |
| A | We have added experimental results of apoptosis assays to the revised manuscript. By TUNEL assay, we found that *Igsf11* deficient spermatocytes were released into the epididymis, where they underwent apoptosis. Data was added to Fig 5F and 5G. |

| Q  38 | 383-386: This sentence contains a very cryptic reference to “SC abnormalities in the mutant nuclei”. What abnormalities? This is the only mention of them and no description or evidence is presented. I did wonder whether there might be a connection between the processes of SC disassembly and chromocenter dissociation as the two processes overlap temporally in late pachytene and diplotene. But that possibility was not explored. It seems there should be some way of characterizing and maybe quantifying the extent of SC disassembly in WT versus mutant. It would be interesting to know if there are correlated defects in these two important disassembly processes in late prophase I. |
| --- | --- |
| A | We have replaced the description of ‘abnormal’ to ‘arrested’. From our results, it appears that SC disassembly and chromocenter dissociation are relatively independent processes from mid-pachytene to mid-diplotene (because SC disassembly is not hindered when chromocenter dissociation was arrested in the absence of IGSF11). It also seems that at around mid-diplotene, a surveillance mechanism is exist to check if these two processes correlated well to each other. Since this is only our speculation without concrete evidence at this moment, we did not discuss much in the text. |

| Q  39 | 392-393 & 409-410: The authors claim that they purified diplotene stage spermatocytes from WT and mutant testes and that the Hi-C results that obtained reflect the situation that pertains at diplotene. But they do not offer any explanation of how they specifically isolated diplotene stage cells or any quality checks to evaluate the level of contamination with other cell types. |
| --- | --- |
| A | Diplotene stage spermatocytes were sorted with the help of the VPHS reporter. Purity of the sorted cells were analysis by meiotic spread (S2C and S2D Fig). Above information has been added to the methods section. |

| Q  40 | Discussion  The main findings are that IGSF11 is required both in Sertoli cells and spermatocytes for meiotic progression in male mice; that mutant spermatocytes arrest at diplotene; and that they fail to undergo disassembly of the chromocenters that cluster pericentric regions of non-homologs during pachytene. The two latter defects constitute an intriguing correlation. To their credit, the authors make no strong claims about a causal relationship, as there is no evidence for one, but they also never make the point that their result is nothing more than an association, which might be useful because readers that skim the paper might assume that one causes the other.  The suggestion that there might be a post-pachytene checkpoint that monitors events like chromocenter disassembly is an interesting one, albeit highly speculative. There are likely a number of alternative explanations that are not explored but a checkpoint seems one plausible mechanism and one that can be tested in a variety of ways. |
| --- | --- |
| A | At this moment, we are still studying the relationship between the disassembly of the chromocenters and arrest at diplotene. We are careful not to prematurely define the potential link without more experimental evidence. We hope that the reviewer would understand our position. |

| Q  41 | A possible weakness with this idea is that it doesn’t have any clear connection with the presumption that IGSF11 mutants impair signaling between somatic cells and spermatocytes implied by the cellular function of IGSF11. If the authors have any ideas about how the germ cell phenotypes might be related to possible soma-germ cell signaling (or germ cell-germ cell signaling), that could strengthen the discussion. |
| --- | --- |
| A | We are still investigating the potential link between IGSF11 signaling between Sertoli cells and germ cells. We hope that we can describe this signaling in more details when we have more experimental evidence in the future. |

**References:**

Alsheimer, M., Baier, A., Schramm, S., Schutz, W., and Benavente, R. (2010). Synaptonemal complex protein SYCP3 exists in two isoforms showing different conservation in mammalian evolution. Cytogenet Genome Res *128*, 162-168. 10.1159/000303341.

Chen, S.R., Hao, X.X., Zhang, Y., Deng, S.L., Wang, Z.P., Wang, Y.Q., Wang, X.X., and Liu, Y.X. (2016). Androgen receptor in Sertoli cells regulates DNA double-strand break repair and chromosomal synapsis of spermatocytes partially through intercellular EGF-EGFR signaling. Oncotarget *7*, 18722-18735. 10.18632/oncotarget.7916.

De Gendt, K., Swinnen, J.V., Saunders, P.T., Schoonjans, L., Dewerchin, M., Devos, A., Tan, K., Atanassova, N., Claessens, F., Lecureuil, C., et al. (2004). A Sertoli cell-selective knockout of the androgen receptor causes spermatogenic arrest in meiosis. Proc Natl Acad Sci U S A *101*, 1327-1332. 10.1073/pnas.0308114100.

Larose, H., Kent, T., Ma, Q., Shami, A.N., Harerimana, N., Li, J.Z., Hammoud, S.S., and Handel, M.A. (2020). Regulation of meiotic progression by Sertoli-cell androgen signaling. Mol Biol Cell *31*, 2841-2862. 10.1091/mbc.E20-05-0334.
